# Magico-religious and social belief of tribals of district Udaipur, Rajasthan

**DOI:** 10.1186/s13002-017-0195-2

**Published:** 2017-12-01

**Authors:** Vandana Singh Kushwah, Rashmi Sisodia, Chhaya Bhatnagar

**Affiliations:** 1 Department of Zoology, University of Rajasthan, Jaipur, India; 2 Department of Zoology, Mohanlal Sukhadia University, Udaipur, Rajasthan India

**Keywords:** Tribes, Magico-religious, Animals, Fidelity level, Ethnomusical, Taboo

## Abstract

**Background:**

Religious beliefs and practices have long influenced human perceptions and uses of nature. Animals in particular play a prominent role in magico-religious practices and provide historical and cultural depth of these relationships. Understanding human-faunal relations is often fundamental to the cause of meaningful wildlife conservation. This study investigates the domestic and wild harvested species used for spiritual and religious purposes among the tribals of six tehsils of Udaipur district.

**Methods:**

The ethnozoological data were obtained by an emic approach, applying different tools such as semi-structured interviews, participatory rural appraisal, key informant interviews, and focus group discussions. The scientific name and species of animals were identified using relevant and standard literature. Present investigation is a part of major concept worked out for study on tribal people and their beliefs. Ethnozoological information was collected by interview of 150 tribals. The questionnaire was prepared in Hindi keeping all parameters in mind. A total of 55 respondents (35 males and 20 females) answered to the magico-religious parameter. The collected data were analyzed through informant fidelity level (FL).

**Results:**

The present study was undertaken to have an insight of the ethnozoological uses of animals prevalent in Bhil, Meena, and Kathodi tribes inhabiting the Udaipur district of Rajasthan. A total of 25 animals used for magico-religious and social purposes were recorded from the study area. Out of the total number of animals, 60% (15) were mammals, 24% (6) were birds, 12% (3) were reptiles, and the rest 4% (1) were the mollusks. Of the total ethnozoological practices, 64% fall in the magico-religious category, 12% in socio-cultural category, 12% in the category of ethnomusical, and 12% in the category of taboos.

**Conclusions:**

The tribal people maintain strong ties with animals at both the material and spiritual level. Study reveals that traditional people depend on local therapies either magico-religious or natural ones in absence of awareness, modern medical facilities, expensive drugs, and poor transportation. However, the use of animal material in such practices is on a decline.

## Background

Animals occupy an important position in culture and religion of traditional societies all over the world. In this context, many of the useful animals are given sacred status because of their important roles in human culture and religion. Examples could be cited of various domestic cow species which are worshipped by the traditional Hindu societies on a regular basis in recognition of their values to mankind [1]. Not only this, even products such as excreta and urine of these animals are smeared on the floor areas of their houses with a belief that these products would sanctify their dwelling. Similarly, certain body parts such as the horn, hide, tail, and feathers of some other species of animals are used in their religious rituals as well. Traditional societies use a number of animals in their magico-religious sphere. Normally, animal parts and products such as the exoskeleton, bone, and glandular secretions are used as sprays, pendants, and amulets to ward off the perceived evil spirits. In many places, it is a common practice to offer animal sacrifices to appease specific deities and ancestral spirits. The sacrificial offerings are regarded as gifts to the deities that are supposed to maintain health and general well-being of those involved in the process [2, 3]. The animal species most frequently used in traditional folk medicine and practices have been recorded in different social-cultural contexts throughout the world [4–14]. Tribal people have faith in animal-based magico–religious therapies to heal a number of diseases and in witchcraft [15, 16]. Ethnic people of Rajasthan are simple, superstitious, God-fearing people with their own customs, traditions, and folklore. They believe in the mystical world which affects health as well as wealth. The literature is scanty about the magico-religious therapies used to cure diseases by tribal people [17–19] of the state. Documentation of such types of beliefs is necessary before it vanishes. Therefore, the present study has been undertaken to know about the role of animals in magico-religious and socio-culture practices in the tribals of remote areas of Udaipur, Rajasthan.

### Study area

**Fig. 1:**
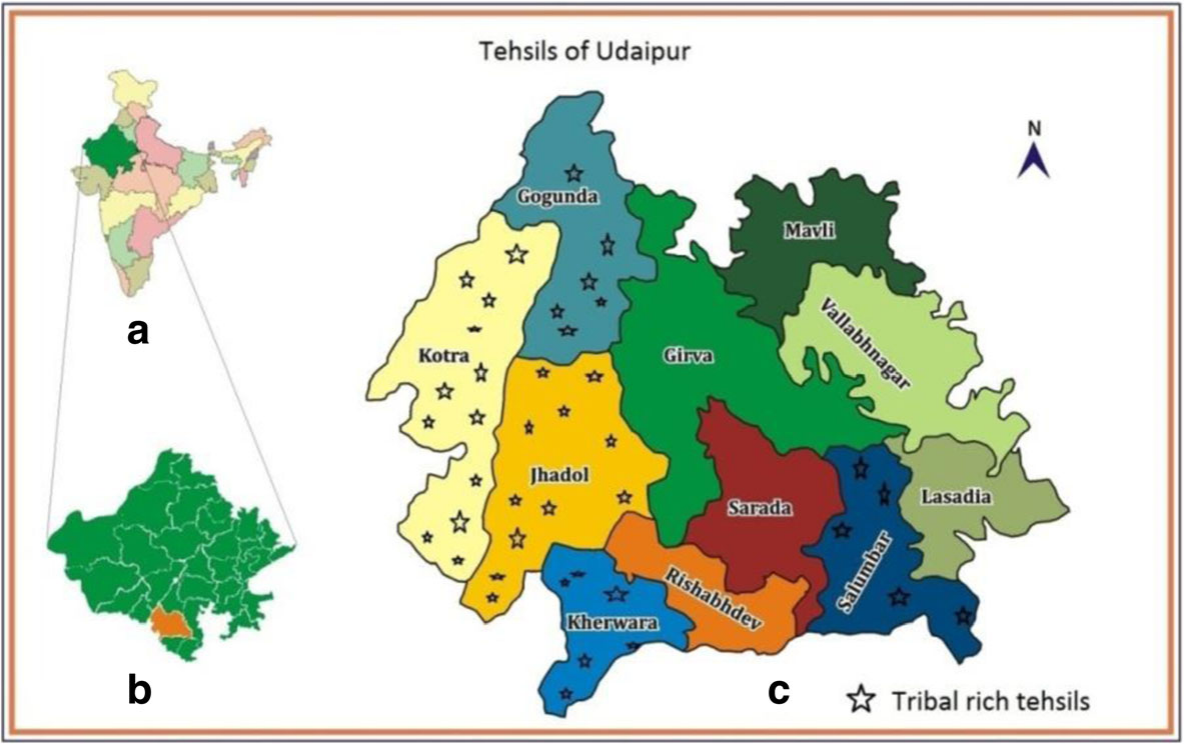
Map depicts the tehsils studied

Rajasthan is located in the north-western part of India. Its geographical location is between 23° 04′ to 30° 11′ and 30° 11′ North and 69° 30′ to 78° 17′ East with the tropic of cancer passing through its southernmost tip. Udaipur district (23° 46′ to 26° 20′ North and 73° 0 East to 74° 35′ East) comprises of 11 tehsils (a tehsil is an administrative division within the district), namely, Kotra, Jhadol, Kherwara, Rishabhdev, Sarada, Salumbar, Lasadia, Vallabhnagar, Mavli, Gogunda, and Girva. Among these, six tehsils (Gogunda, Salumbar, Kotra, Jhadol, Girva, and Kherwada) are rich in tribal population, and therefore, these were the focus of the study (Fig. 1).

## Methods

### Design of study

A general idea about the district-wise distribution of these tribes was obtained from the tribal map of Rajasthan. These tribal-rich areas were considered as study area and were the sites of survey. The ethnozoological data were obtained by an emic approach, through the following tools:

- Semi-structured interviews: These are qualitative methods of enquiry that combines a pre-determined set of open questions (questions that prompt discussion) with the opportunity for the interviewer to explore a particular objective.
- Participatory rural appraisal (PRA): It is a suitable method used to gather information on village resources and their distribution, cropping calendar, festival calendar, and animal uses during different festivities.
- Key informant’s interviews: These are qualitative in-depth interviews with people who have specialized knowledge in a desirable objective.
- Focus group discussions (FGD): It is a good way to bring together people from similar backgrounds or experiences to discuss a specific topic of interest.

Out of the 150 participants, the ethnozoological information was obtained from 55 respodents (35 male and 20 female). The age of the informants was between 25 and 55 years, with the percentage of the younger generation being only 10%. In each district, the same tribes were interviewed as far as possible to get elaborate information and regional differences, if any, regarding the relation of tribes and role of animals in magico-religious rituals. The interviews were conducted in the different clans of tribes which included Bhil, Meena, and Kathodi. Ethnozoological data from the key informants and from other knowledgeable people of the village were gathered by method given by suitable method [20]. The scientific name and species of animals were identified using relevant and standard literature [21, 22].

### Data analysis

Fidelity level (the percentage of respondents claiming use of a particular animal species for same ritual) of the data obtained was calculated for the most frequently reported animal use value:

FL (%) = Np × 100/*N*,

where Np is the number of respondents that claim the use of a species in particular ritual and *N* is the number of respondents that use the animals for magical ritual [23]. The FL is from 1 to 100%; value close to 100 means high use of a particular animal species by large number of people, while a low value shows that the respondents disagree on that species to be used in the magical treatment.

## Results and discussion

**Fig. 2:**
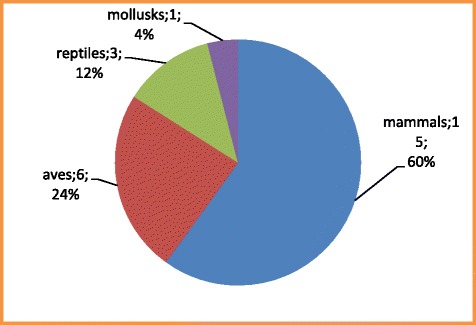
Number of animal species used for magico-religious and other purposes per taxonomic category in tehsils of Udaipur, Rajasthan

The results obtained indicate that tribals use a variety of animals in their rituals. Out of these, 60 (15) were mammals, 24% (6) were birds, 12% (3) were reptiles, and the rest 4% (1) was a mollusk (Fig. 2)**.** Of the total uses, 64% fall in the magico-religious practices, while 12% each in the category of making musical instruments with the skin, for socio-culture practices (viz., making ornaments, garments, and weapons) and as taboos. An understanding of the cultural, social, and traditional role of these animals is fundamental for establishing management plans directed towards the sustainable use [19].

### Animals in a magico-religious sphere

Magic is the imperative part of tribal life of Rajasthan. It is employed in curing various diseases thought of to be due to evil spirits. The magico-religious rituals are performed by the priest locally as “bhopa.” The magico-religious beliefs of tribals as observed in the present study are summarized in Table 1.

**Table 1:** Magico-religious animals and their major uses with their fidelity Level

S. no.	Scientific name	Common name	Tribal names	Parts and their uses	No. of respondents claimed (*n*)	Fidelity level (FL)	Tribes
1	*Athene brama* (Temminck, 1821)	Spotted owlet	Chhibra	Charms are made out of its bones, and it is believed that these drive evil spirits away	49	89%	Meena
2	*Hyaena hyaena* (Linnaeus, 1758)	Striped hyena	Hiyaliya	Fumes of burnt flesh to be inhaled by the sick for these are thought to drive evil spirits away	12	21%	Meena
3	*Geochelone elegans* (Schoepff, 1795)	Indian star tortoise	Kasba	Skeleton and is believed that these drive evil spirits away.	23	41%	Bhil, Meena
4	*Corvus macrorhynchos* (Wagler, 1827)	Jungle crow	Dodh Kagda	Charms are made out of bones, and it is believed that these drive evil spirits away.	9	16%	Meena
				Eggs are eaten for telepathy power	18	33%	Meena
5	*Hemidactylus frenatus* (Schlegel, 1836)	Common lizard	Kangetia	Tail tied in waist and it is believed that this kept disease away	11	20%	Meena
6	*Canis aureus* (Linnaeus, 1758 ^[^)	Jackal	Gadarda	Charms are made out of bones, and it is believed that these drive evil spirits away	9	16%	Meena
7	*Panthera tigris tigris* (Linnaeus, 1758)	Tiger	Vanraj	Charms are made out of nails, and it is believed that these drive evil spirits away	53	96%	Meena, Bhils, and Kathodi
8	*Melursus ursinus* (Shaw,1791)	Sloth bear	Rechha	Charms are made out of nails and bone, and it is believed that these drive evil spirits away	47	85%	Kathodi
9	*Ocyceros birostris* (Scopoli, 1786)	Indian gray hornbill	Dhanada	Charms are made out of bill, and it is believed that these drive evil spirits away	43	78%	Meena, Bhils
10	*Sus scrofa* (Linnaeus, 1758)	Wild boar	Sukar	Charms are made out of bones, and it is believed that these drive evil spirits away	8	14%	Meena
11	*Pteropus giganteus* (Brünnich, 1782)	Bat	Chakchoondar	Charms are made out of bones, and it is believed that these drive evil spirits away	17	31%	Meena
12	*Crocodylus palustris* (Lesson, 1831)	Crocodile	Magur	Bones are used to drive evil spirits away	7	13%	Meena, Bhils
13	*Hystrix indica* (Kerr, 1792)	Porcupine	Sehli	Its spines are kept in the house with a belief that they drive off evil spirits and disease-causing elements	29	53%	Bhil, Kathodi
14	*Bos taurus indicus* (Linnaeus,1758)	Cow	Gau	Spray of a urine to drive evil away	55	100%	Meena, Bhils, and Kathodi
15	*Panthera pardus fusca* (Meyer, 1794)	Leopard	Cheetra	Charms are made out of nails, and it is believed that these drive evil spirits away	50	91%	Meena, Bhils, and Kathodi
16	*Pavo cristatus* (Linnaeus, 1758)	Peacock	Moriyo	Feathers are used for performing magical ritual called jaada to drive evil spirit away	38	69%	Meena, Bhils

(FL below 0 = least efficiency, 100 = highest efficiency)

In the present study, the highest FL value calculated was for *Bos taurus indicus* (100%) followed by *Panthera tigris tigris* (96%). The lowest was for *Crocodylus palustris* (13%). It was observed that the urine of cow (*Bos taurus indicus*) had the highest FL (*N* = 55, 100%) followed by the claws of tiger (*Panthera tigris tigris* with *N* = 53, FL = 96%) that were used to drive evil spirits away. The bones of wild boar (*Sus scrofa*, *N* = 8, 14%) and crocodile (*Crocodylus palustris*, *N* = 7, 13%) had the lowest FL. This low percentage may reflect a low level of use of animal for musical instruments, taboos, and socio-culture practices, or it may be related to a decrease in transmission of this type of knowledge to the younger generations.

Animals and their parts are also reported to be used as charms in treating different magico-religious rituals [24, 25]. Animal parts and products such as bones and claws are used in making “charms” to prevent from “evil and disease-causing elements,” and these charms are tied around the neck, arm, or waist [26–32]. A different category of magical belief is the evil eye or commonly called “nazar.” Tribal people think that the evil eye or nazar is caused by black magic performed by enemy leading to illness especially among children. For protection from evil eye, amulets, charms, and pendants are made from animal parts and tied around the neck or waist. These charms are locally called “Thabij” and are made of hard parts of the animal such as its endo- or exoskeleton. Similar relationship of the tribal people with the animals at the spiritual/cultural level is also reported from different parts of the developing world [12, 19, 33–36].

The animals or their parts used by tribals for various purposes such as making instruments, weapons, and garments are presented in Table 2.

**Table 2:** Socio-cultural uses of animals in tribes

Uses		Animal used	Part of animal	Tribal clans		
				Bhil	Meena	Kathodi
Caps		*Petaurista philippensis* (Elliot, 1839)	Whole body	No	No	Yes
Weapons		*Accipiter badius* (Gmelin, 1788)	Feathers	Yes	No	No
Ornaments		*Pavo cristatus* (Linnaeus, 1758)	Feathers	Yes	No	No
		*Cypraea annulus* (Linnaeus, 1758)	Shell	Yes	No	No
Garments		*Cypraea annulus* (Linnaeus, 1758)	Shell	Yes	No	No
Musical instruments	Dholak	*Capra hircus* (Linnaeus, 1758)	Skin	Yes	Yes	No
		*Semnopithecus entellus* (Dufresne, 1797)	Skin	No	No	Yes
	Thapa	*Bos taurus* (Linnaeus, 1758)	Horns	No	No	Yes

### Ethnomusical animals

The dried skin of *Capra hircus* (goat) is used in making a kind of musical instrument called “Dolak” by the Bhil community. The Kathodi community use the skin of *Semnopithecus entellus* (Hanuman langur) for the same purpose (Table 2). A famous musical instrument of Kathodi called Thapa is made with horns of *Bos taurus*. Similar uses of animals (or parts) in ethnomusical instruments have also been reported from Nepal and Cameroon [32, 37].

### Animal products and their use in socio-cultural practices

The products of different animals used in different socio-cultural activities of the tribes as observed in the present study are described below:

1. Ornaments: The feathers of *Pavo cristatus* (peacock) are used as earrings and decorating masks by Bhils who wear them while performing the famous traditional “gavri” dance (Table 2). Similar use of peacock feathers is observed in Santhals of West Bengal, India [38]. They also decorate their cattle with these feathers. Different types of jewelry made with *Cypraea annulus* (locally called cowrie) are worn by the tribals of the study area on different parts of the body. Teeth, feathers, and skin of various animals are used in making jewelry in Cameroon, Africa [37].
2. Garments: Traditional dance dresses are decorated with *Cypraea annulus* (white shell) by Bhils (Table 2).
3. Cap: It is used by children of Kathodi tribes and is made up of the skin of *Petaurista philippensis* or giant flying squirrel (Table 2). Similar caps made up of tail hair of yak have also been reported from Arunachal Pradesh, India [33].
4. Weapons: In Bankura district of West Bengal, horns of cow and buffalos are used as weapons [38]. No such use of any animal part is reported in the present study. Feathers of *Accipiter badius* (shikra) are used to decorate the rear end of arrows especially by Bhils (Table 2). Similar use of skin and hair of wild goat in decoration of tools is reported from Arunachal Pradesh, India [33].

### Taboos

In Bhil community, killing of squirrel (*Funambulus pennanti*, Wroughton, 1905) or house cat (*Felis domesticus*, Linnaeus, 175) is prohibited and it is believed that if someone kills the squirrel and cat, they will go to hell. Sarus crane (*Grus antigone*, Linnaeus, 1758) killing is a big taboo in all the three clans (Bhils, Meena, and Khatodi), and they believe that the handicapped child will be born in the killer family. Fishing is also tabooed in breeding season by all clans. Similar kinds of taboos have been reported from Nepal [39]. It is a taboo to kill the female animal (especially gravid female) among the inhabitants in the northern Trans Himalayan area of the Nepal [39]. These kinds of taboos regarding killing of animals directly or indirectly help in conserving the animals. From the ancient time, these activities of tribal people have formed an integrated resource conservation method. Four types of taboos (habitat, species-specific, method, and segment) have been reported from Africa [37].

Literature also suggested that a variety of animals forms an integral part of cultural and religious festivals and ceremonies, some of which seek to promote the good health of local people and their communities [35]. Some previous studies indicate that while performing of certain rituals and festivals, a species may also be sacrificed [40] to bring good health and prosperity. However, no such rituals of animal sacrifices were observed in the present study.

## Conclusion

The present study is based on the deep discussions with the tribal people and their beliefs in magico-religious treatment to cure a disease and maintain a general well-being. Indigenous people of the Udaipur district have the belief that diseases originate due to supernatural forces and they seek treatment through magico-religious practices. On the direction of Bhopas, the tribals offer prayers and sacrifices to appease the supernatural power, which may be responsible for the disease. The study reveals that traditional people depend on local therapies either magico-religious or natural due to the lack of awareness, modern medical facilities, expensive drugs, and poor transportation. The animal products are also common for varied uses, viz., ornaments, garments, musical instruments, weapons, and charms. These commonly used articles are now being transferred from one generation to another. The increase in awareness among tribals and legal consequences have refrained them from sacrificing animals. Thus, in modern context, these tribals are now conscious towards conserving the animals rather than destroying them.

### Recommendations

On the basis of the present study, it is recommended that there should be a regular monitoring and contact with the tribals regarding their beliefs. They need to be educated and made aware of the value of animals and the great loss incurred if a species disappears.
